# Editorial: Improving *in vitro* modeling of human brain with future brain organoids

**DOI:** 10.3389/fnmol.2022.997119

**Published:** 2022-08-09

**Authors:** Serena Barral, Yangfei Xiang, Fikri Birey

**Affiliations:** ^1^Developmental Neurosciences, Zayed Centre for Research Into Rare Disease in Children, Great Ormond Street (GOS)-Institute of Child Health, University College London, London, United Kingdom; ^2^School of Life Science and Technology, ShanghaiTech University, Shanghai, China; ^3^Department of Human Genetics, Emory University, Atlanta, GA, United States

**Keywords:** brain organoids, assembloids, high-throughput, neural network, neurodevelopmental, neurological disorders, therapeutic strategies

Progress in understanding human brain biology and related disorders has been remarkably slow due to limited access to functional brain tissue, hampering the development of effective therapies. In response to this challenge, advances in three-dimensional (3D) culture systems over the last decade have led to the development of human pluripotent stem cell (hPSC)-derived brain organoids that have revolutionized the study of the human central nervous system (CNS) and neurological disorders. To date, 3D human brain organoids have been used in numerous efforts to model increasingly complex aspects of human brain development and physiology in health and disease (Paşca, 2018; Velasco et al., 2020; Kelley and Paşca, 2022).

A flurry of protocols has been developed to establish 3D human stem cell-derived models that resemble various human brain regions, providing unique advantages over conventional 2D cultures; for example, in contrast to their 2D culture counterparts, 3D human brain organoids can be maintained in culture for months to years, reach later stages of development, and thus better recapitulate human brain physiology in normal and disease states (Gordon et al., 2021). The latest state-of-art protocols including brain region-specific organoids and assembled multi-region brain organoids (i.e., “assembloids”) are now employed in conjunction with single-cell transcriptomics, electrophysiology and live cell imaging, allowing for the monitoring and assessment of developmental phenomena that have historically been inaccessible for functional investigations (Birey et al., 2017; Xiang et al., 2019; Miura et al., 2022). Yet, several outstanding questions remain: How closely can human brain development and physiology be recapitulated *in vitro*? How robust and reproducible are these *in vitro* generated models? What are the standards and best practices in experimental design? Questions of “fidelity” and “reproducibility” now sit at the root of brain organoid-based modeling efforts and work in this direction constitutes a priority to better model human brain development, understand disease states, and develop novel therapeutic strategies.

This Research Topic aims to explore recent advances in human *in vitro* models based on 3D human brain organoids with special emphasis on modeling various disease states and therapeutic approaches:

A major limitation inherent in organoid models is the inter-organoid variability, which leads to underpowered measurements of physiological processes and limited capabilities for testing efficacy in compound screens (Quadrato et al., 2017; Hofer and Lutolf, 2021). Here, Renner et al. developed an automated fluorescence-based workflow to evaluate the toxicity of a library of compounds in midbrain organoids generated *via* an automated workflow. Their work highlights the importance of highly reproducible and standardized culture conditions to properly assess single-cell responses to different compounds and enable cell type-specific therapeutic strategies. This is further discussed in depth by Fan et al., who summarize recent developments in toxicology studies using organoid models. Moreover, the authors explore current limitations highlighting required technological improvements aimed at fully recapitulating organism-level responses and providing cell types/tissue structures derived from non-ectodermal germ layers such as vasculature.

Cellular composition and network complexity are major benchmarks of “functional fidelity” of brain organoids. This topic is discussed by Zourray et al. which address the importance of multi-cellular systems to properly reproduce neuronal network maturation in cortical organoids. Recent protocols aiming to reproduce multiple regions of the human brain have provided a major improvement in modeling different cortical networks. Zourray et al. discusses novel protocols integrating microglial cells, a major cell type critical in the control of neuronal synaptic maturation, aimed at improving the electrophysiological maturity observed in the human brain organoids. Overall, the authors highlight the current lack of standardized electrophysiological analysis, suggesting the need for a rigorous workflow to improve the reproducibility of electrical recording of neural networks in cortical organoids and assembloids.

As the fidelity and reproducibility of human brain organoid protocols improve, so will their ability for robust disease modeling using patient-derived cells, which remains one of the core promises of hPSC-based models of human brain organogenesis (Kelley and Paşca, 2022). Several critical advances in modeling human brain disorders have been extensively discussed in three reviews of this Research Topic. Borges et al. and Deb and Bateup report recent progress in the study of phenylketonuria and neurodevelopmental disorders, respectively. Borges et al. suggests how the use of multi-organ organoid-based systems would benefit the study of complex disorders affecting different biological pathways not only restricted to the brain. Deb and Bateup suggest that genome engineering of hPSCs will improve standard analysis of somatic mutations acquired during brain development and how they alter normal maturation of brain structures. More in-depth studies will allow dissecting the contribution of subcellular organelles to neural development and neuronal maturation as reviewed by Romero-Morales and Gama, which focuses on the role of mitochondrial fitness in brain development. Ultimately, Lange et al. argue that brain organoids allow first-in-human testing of therapeutic strategies and accelerate their clinical translation, and give an overview of how a recent therapeutic approach, anti-sense oligonucleotides, can be implemented in human brain organoids.

Rapidly evolving human brain organoid-related technologies represent a new frontier in neurobiology and stem cell biology, facilitating the modeling of increasingly sophisticated aspects of human brain development and disease at an unprecedented scale and resolution. In future, one could envision novel protocols to implement various bioengineering and synthetic biology techniques to enable spatial control over patterning cues, multi-omics approaches to unbiasedly survey the cross-modal sources of variability at the hPSC ground state, innovate cell culturing and “organ-on-a-chip” platforms to incorporate multiple non-ectodermal cell types in brain organoids. These efforts will in parallel enable cutting-edge assays such as high-throughput pharmacology and CRISPR screens, longitudinal monitoring of network activity through high-density multi-electrode arrays, and multi-photon imaging.

Ultimately, human brain organoids will help bring us one step closer to understanding the principles that guide human brain assembly and enable us to accelerate clinical translation of therapeutic approaches for brain disorders.

## Author contributions

SB, YX, and FB drafted and edited the manuscript. All authors approved the submitted version.

## Funding

SB was funded by the Great Ormond Street Hospital Children's Charity and partially funded by the NIHR GOSH BRC. YX received funding form the National Key Research and Development Program of China (2021YFF1200800). FB received funding from National Institute of Mental Health (K99 MH119319).

## Conflict of interest

The authors declare that the research was conducted in the absence of any commercial or financial relationships that could be construed as a potential conflict of interest.

## Publisher's note

All claims expressed in this article are solely those of the authors and do not necessarily represent those of their affiliated organizations, or those of the publisher, the editors and the reviewers. Any product that may be evaluated in this article, or claim that may be made by its manufacturer, is not guaranteed or endorsed by the publisher.
